# Chromosome 2p14 Is Linked to Susceptibility to Leprosy

**DOI:** 10.1371/journal.pone.0029747

**Published:** 2012-01-06

**Authors:** Qing Yang, Hong Liu, Hui-Qi Low, Haifeng Wang, Yongxiang Yu, Xi'an Fu, Gongqi Yu, Mingfei Chen, Xiaoxiao Yan, Shumin Chen, Wei Huang, Jianjun Liu, Furen Zhang

**Affiliations:** 1 Shandong Provincial Institute of Dermatology and Venereology, Shandong Academy of Medical Science, Jinan, Shandong, China; 2 Shandong Provincial Hospital for Skin Diseases, Jinan, Shandong, China; 3 Shandong Provincial key lab of Dermato-venereology, Jinan, Shandong, China; 4 Shandong Provincial Medical Center for Dermatovenereology, Jinan, Shandong, China; 5 Department of Human Genetics, Genome Institute of Singapore, Singapore, Singapore; 6 Shanghai-MOST Key Laboratory of Health and Disease Genomics, Chinese National Human Genome Center at Shanghai, Shanghai, China; Institute of Infectious Diseases and Molecular Medicine, South Africa

## Abstract

**Background:**

A genetic component to the etiology of leprosy is well recognized but the mechanism of inheritance and the genes involved are yet to be fully established.

**Methodology:**

A genome-wide single nucleotide polymorphism (SNP) based linkage analysis was carried out using 23 pedigrees, each with 3 to 7 family members affected by leprosy. Multipoint parametric and non-parametric linkage analyses were performed using MERLIN 1.1.1.

**Principal Findings:**

Genome-wide significant evidence for linkage was identified on chromosome 2p14 with a heterogeneity logarithm of odds (HLOD) score of 3.51 (rs1106577) under a recessive model of inheritance, while suggestive evidence was identified on chr.4q22 (HLOD 2.92, rs1349350, dominant model), chr. 8q24 (HLOD 2.74, rs1618523, recessive model) and chr.16q24 (HLOD 1.93, rs276990 dominant model). Our study also provided moderate evidence for a linkage locus on chromosome 6q24–26 by non-parametric linkage analysis (rs6570858, LOD 1.54, p = 0.004), overlapping a previously reported linkage region on chromosome 6q25–26.

**Conclusion:**

A genome-wide linkage analysis has identified a new linkage locus on chromosome 2p14 for leprosy in Pedigrees from China.

## Introduction

Leprosy is a chronic infectious disease caused by *Mycobacterium leprae*. It affects the skin and peripheral nerves and can cause irreversible impairment of nerve function and consequent chronic disabilities [1]. According to the World Health Organization, the global registered prevalence of leprosy at the beginning of 2010 stood at 211,903 cases. Infection is necessary for the onset of disease, but only a small proportion of infections lead to clinically recognizable lesions [2]. Host genetic factors have been implicated in susceptibility to leprosy in studies of familial clustering [3], studies of twins [4] and complex segregation analyses [5], [6]. Various genes (*HLA-DR* [7], [8], *PARK2/PACRG* [9], *LTA* [10], *TLRs* [11], [12], etc.) and genomic regions (10p13 [13], 6q25–26 [14], 6p21 [14], 17q11–q21 [15], 20p13 [16], etc.) of human genome have been associated with or linked to leprosy (or a particular clinical form of leprosy) by candidate gene association studies or genome-wide linkage analysis. Nonetheless, few of these results have been replicated in different populations. These results suggest that susceptibility to leprosy is polygenic, with a high degree of heterogeneity among different populations. We recently reported a genome wide association study (GWAS) of leprosy and identified significant associations between single nucleotide polymorphisms (SNPs) in the genes *CCDC122*, *C13orf31*, *NOD2*, *TNFSF15*, *HLA-DR*, and *RIPK2* and a trend toward an association with a SNP in *LRRK2*. Five of these genes encode proteins involved in the innate immune response [17]. Here, we present a genome-wide SNP-based linkage analysis of 23 multiplex families, each with at least 3 patients with leprosy.

## Results

**Figure 1:**
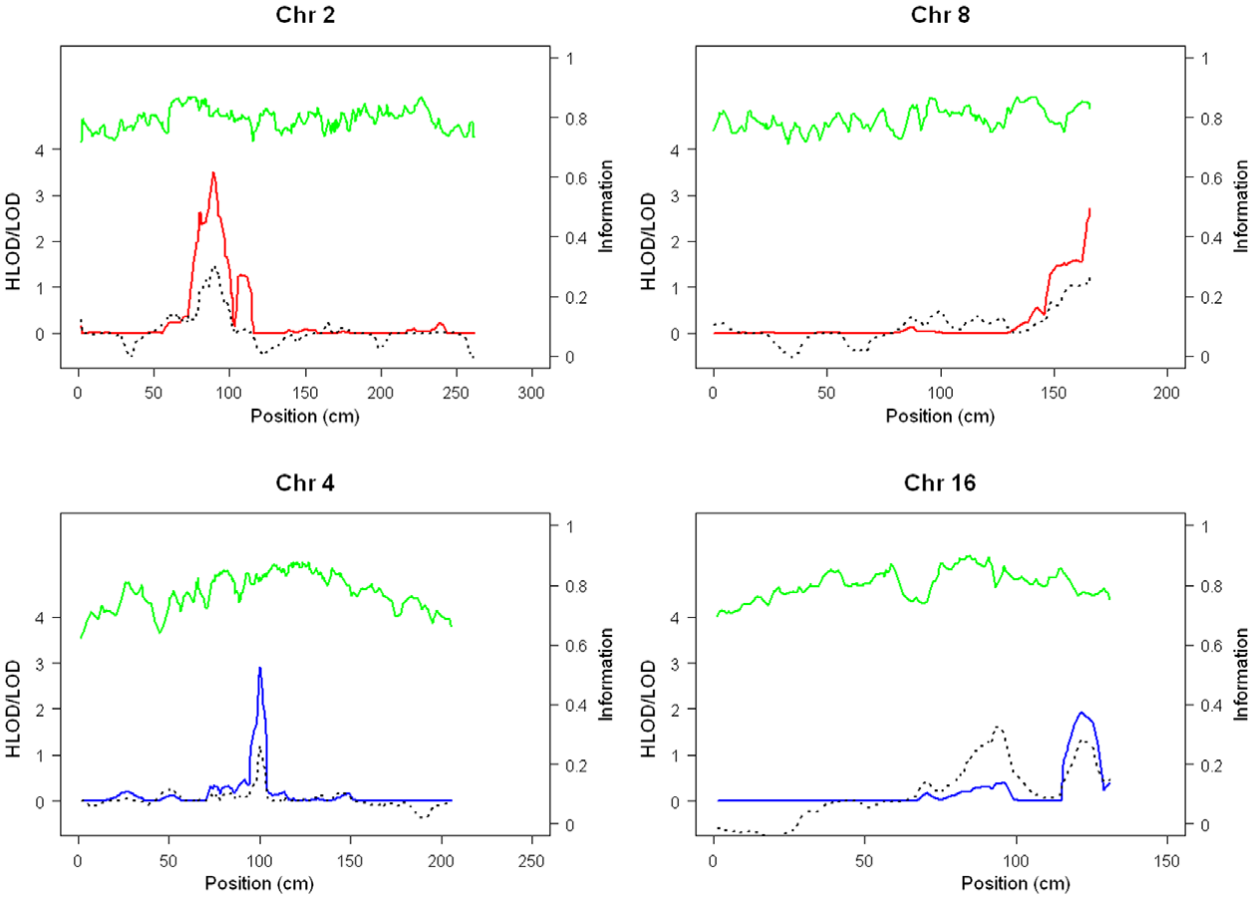
Genome-wide linkage results. Genomic position is shown on the horizontal axis; HLOD(parametric Model)/LOD(non-parametric model) score on the left vertical axis.; Information content on the right vertical axis. Red line indicates results under a recessive model; blue line indicates a dominant model, dashed line indicates nonparametric analysis, and green line indicates information content.

A total of 82 patients and 16 unaffected individuals from 23 multi-case leprosy families were genotyped in the present study. After quality control filtering,the linkage analysis was carried out using 5525 autosomal SNPs. The most noticeable results of the genome-wide linkage analysis were summarized in Table 1 and shown in Figure 1. The maximum HLOD score of 3.51 was detected on chromosome 2p14 at rs1106577 under a recessive model of inheritance with a full penetrance. The critical region extends from rs890478 to rs758062 on 2p13.3–14, including 16 markers on 2p13.3–14. As shown in Supplementary Table S1, 49 SNPs show supportive evidence (HLOD>1) for the linkage on this locus. Approximately 45% of families were consistent with linkage to this region. Varying the penetrance rate has little effect on the linkage results; the maximum HLOD score was still above 3.0 assuming a penetrance of 0.5 at the same locus.

**Table 1:** Parametric and non-parametric linkage analysis of leprosy families.

Chr. No.	Position (cm)of peak HLOD score	marker	Parametric analysis model	HLOD (α)Penetrance = 1	HLOD (α)Penetrance = 0.5	Non-parametricLOD (P value)
2	89.24	rs1106577	recessive	3.51 (0.45)	3.01 (0.45)	1.48 (0.005)
8	166.01	rs1618523	recessive	2.71 (0.38)	2.74 (0.41)	1.22 (0.009)
4	100.28	rs1349350	dominant	2.92 (0.44)	2.79 (0.44)	1.21 (0.009)
16	121.92	rs276990	dominant	1.93 (0.35)	1.86 (0.35)	1.37(0.006)
6	150.56	rs6570858	dominant	0.89 (29.4)	1.00 (0.32)	1.54(0.004)

To evaluate the empirical significance of our linkage results, we conducted a simulation analysis to evaluate the significance of our results using the criteria proposed by Lander and Kruglyak [18]. Our simulation analysis has indicated (Supplementary Table S2) that for parametric analysis under a recessive model with a full penetrance, the threshold for genome-wide significant linkage is 3.148 and 0.948 for suggestive linkage. Under a dominant model, the thresholds were 3.033 for genome-wide significant linkage and 0.869 for suggestive linkage. For the non-parametric linkage analysis, the thresholds are 3.11 for genome-wide significance and 0.88 for suggestive linkage. Based on the simulation results, the linkage evidence for the locus on chromosome 2p14 reached the genome-wide significance.

Linkage was also identified on chr.4q22.2–22.3 (maximum HLOD 2.92,rs1349350,under dominant model), chr.8q24.3(maximum HLOD 2.71, rs1618523, under recessive model), chr.16q24.1–q24.2 (maximum HLOD 1.93, rs276990,under dominant model) with the full penetrance, and chr.6q24–26 (maximum LOD 1.54, rs6570858, p = 0.004, by non-parametric linkage analysis). When the penetrance was varied in the parametric analysis, the maximum HLOD score of these regions changed slightly. According to our simulation results, these linkage results are only suggestive. The linked regions identified by parametric analysis were also supported by nonparametric analysis (Table 1). The locus on chromosome 6q24–26 overlapped a previously reported linkage region on 6q25–26 [14]. The parametric linkage analysis under a dominant model also supports the linkage within the locus with suggestive evidence.

## Discussion

In this study, we performed a genome-wide linkage analysis using a high-density whole-genome linkage array with the median distance between SNP markers of 441 kb and identified a novel susceptibility locus for leprosy on chromosome 2p14 under a recessive model of inheritance. Suggestive evidence of susceptibility loci were found on chromosome 4q22 and 16q24 under a dominant model, chromosome 8q24 under a recessive model and chromosome 6q24–26 by non-parametric analysis. Not all of our pedigrees showed linkage in each of these chromosome regions and this suggests potential genetic heterogeneity among different leprosy families. Our results suggest the presence of multiple genetic variants predisposing to leprosy under different modes of inheritance. The linked regions were supported by parametric (either under dominant or recessive models) as well as non-parametric linkage. Parametric linkage analysis is more powerful than non-parametric methods for detecting linkage, with differences in power determined by the true underlying model and linkage information content. Although there is uncertainty about the true penetrance of leprosy, varying the disease penetrance has little impact on our linkage results, suggesting that the linkage results are stable and no depending on the penetrance. This is expected, because all the linkage analyses were performed by treating all the family members without disease phenotype as ‘unknown’.

Previous linkage studies using microsatellite markers have identified several linkage loci on chromosome 10p13 [13], 6q25–26 [14], 6p21 [14], 17q11–q21 [15], and 20p13 [16]. Our results provide further supporting evidence for the linkage within the 6q25 region, though the linkage evidence from the nonparametric analysis was moderate with a max LOD score of 1.54 (p = 0.004). SNPs within the shared promoter region of the *PARK2* and *PCARG* genes on this locus have been identified to be associated with leprosy susceptibility in two ethnically distinct populations Vietnamese and Brazilian [9]. Our study does not provide evidence for the previous reported linkages on other loci. The inconsistent results across the different ethnic groups could be the result of genetic heterogeneity of leprosy between populations or the limited power of our study.

There seems to be little overlap between the regions/loci identified in the present linkage study of leprosy families and the ones revealed by our previous GWAS of unrelated leprosy cases and controls. The discordant results are not surprising, since the loci identified by the current linkage and the previous GWAS analyses may be different. The current analysis is more likely to identify variants with relatively strong genetic effects (high penetrance) and thus causing familial aggregation where multiple family members were affected with the disease. Such linkage loci may harbor relative rare variant, potentially showing allelic heterogeneity across families, which would require a direct re-sequencing analysis to uncover. In contrast, the GWAS analysis is more likely to identify common genetic variants with lower penetrance whose genetic effect are too moderate to cause familial aggregation of the disease and thus be detected by linkage analysis with the current sample size. Linkage and association analyses are therefore complement and both needed to reveal the full spectrum of genetic risk variants for leprosy.

There are several limitations to our study. First, the size of the sample is modest. Replication of our results in independent samples (especially in different ethnic groups) will be essential. Second, we concentrated our efforts in large leprosy pedigrees with a possible stronger genetic component. Thus, these results might have overestimated the magnitude of the effect of these loci in general population. Third, while it is possible that MB and PB forms of leprosy have some different predisposing genetic factors, it is not feasible to conduct subgroup analysis in this study due to the sample size. Our study of all the pedigrees together may help to identify genetic factors that are shared by MB and PB. Notwithstanding these limitations, our study provides strong genetic evidence of a novel susceptibility locus for leprosy on chromosome 2p13.3–14 and suggested several other regions of potential interest.

There are a number of genes of potential interest within 2p14 region that are involved in innate immune response, particularly in endocytosis process, including *CLEC4F*, *CD207*, *ATP6V1B1*, *PPP3R1*, *KIAA1048*, *ANXA4* and *AAK1*. These results may help guide further studies on leprosy. The analysis of additional leprosy pedigrees, in addition to fine-mapping and/or resequencing to identify susceptibility genes and functional variation within the linkage regions will further validate these findings. Elucidation of the genetic factors that influence susceptibility to leprosy may provide new insight into the prevention and control of the disease.

## Materials and Methods

### Sample collection

A collection of 23 multiplex families with 3 to 7 family members affected with leprosy was enrolled from Shandong, Jiangsu and Yunnan provinces, including 13 families of Chinese Han, 5 of Miaozu, 2 of Yizu, 1 of Daizu, 1 of a mixed Han and Yizu and 1 of a mixed Han and Baizu. The diagnosis of leprosy was based on medical records stored in local leprosy control institutions and clinical assessments at the time of blood taken (looking for evidence of leprosy such as claw hand, lagophthalmos or foot drop, etc). Demographic characteristics, clinical subtypes and age at onset of the disease were also collected from medical records. The classification of the patients was based on clinical and histological criteria [19]. Patients were classified into two clinical subtypes: multibacillary (MB) form including patients with lepromatous(LL), borderline lepromatous (BL) and borderline(BB) leprosy and paucibacillary (PB) form including patients with borderline tuberculoid (BT) and tuberculoid (TT) leprosy. In all, 17 families (73.9%) contained both MB and PB affected individuals, 4 families (17.%) contained only MB affected individuals and the remaining 2 families (8.7%) had only PB affected individuals. Characteristics of the families are summarized in Table 2, and the pedigree structures of these families and clinical subtype of each patient are shown in Supporting Information S1. All subjects gave written informed consent to participate in the study. The protocol was approved by the Ethical Committee of the Shandong Provincial Institute of Dermatology and Venereology.

**Table 2:** Family structures for 23 leprosy families.

Affected patients per family	number of families	Number of patients	Unaffected individuals	Number of families with PB only	Number of families with MB only	Number of families with Both PB and MB
3	15	45	6	2	3	10
4	5	20	5	0	1	4
5	2	10	2	0	0	2
7	1	7	3	0	0	1
Total	23	82	16	2	4	17

### Genotyping

EDTA anticoagulated venous blood samples were collected from all the participants. Genomic DNA was extracted from peripheral blood lymphocytes by standard procedures using Flexi Gene DNA kits (QIAGEN, Germany). Genomic DNA samples were diluted to working concentrations of 50 ng/µl for genotyping analysis. DNA samples were surveyed for quality both by a Nanodrop Spectrophotometer (ND-1000) and the electrophoresis assay. Approximately 200 ng of genomic DNA was used for genotyping analysis. Briefly, each sample was whole-genome amplified, fragmented, precipitated and resuspended in appropriate hybridization buffer. Denatured samples were hybridized on prepared Illumina Linkage-12 Human DNA Analysis Kit (Illumina, San Diego, USA). After hybridization, the BeadChip oligonucleotides were extended by a single labeled base, which was detected by fluorescence imaging with an Illumina Bead Array Reader. Normalized bead intensity data obtained for each sample were loaded into the Illumina BeadStudio 3.3 software, which converted fluorescence intensities into SNP genotypes.

### Linkage analysis

The genome-wide linkage analysis was performed by using a total of 6090 SNP markers, having average 0.58 cM genetic map spacing and average 441 kb physical map spacing. The patterns of disease transmission did not support an X-linked mode of inheritance in the leprosy pedigrees, the X-chromosome was not analyzed. SNPs with a call rate less than 90% or with cluster plots that did not show clear separation of the three genotype clusters were excluded. A total of 5525 autosomal SNPs were retained in the linkage analysis. The average minor allele frequency (MAF) of the SNPs was 0.276.

Multipoint parametric and non-parametric linkage analyses were performed via the program of MERLIN version 1.1.1 [20]. Due to the uncertainty of inheritance model underlying the disease phenotype, parametric linkage analysis was performed by assuming both a dominant and a recessive model of inheritance with various penetrances of 1.0, 0.8, 0.5 and 0.3 and a fixed disease prevalence of 0.0001. All the parametric linkage analyses were performed in a affected only fashion where all the individuals without disease phenotype were treated as “unknown”. Mendelian inconsistencies in the genotype data were investigated with Pedcheck version 1 and improper genotypes were set to “missing” before the linkage analysis. Because leprosy is assumed to be a complex disease and probably arise from multiple heterogeneous loci, we report heterogeneity LOD (HLOD) scores that can more accurately reflect the true position of a linkage peak and have been shown to be more powerful than homogeneity LOD scores and model-free methods under conditions of heterogeneity [21]–[23]. An estimate of α, which represents the proportion of pedigrees consistent with linkage at a specific locus, was also calculated. Nonparametric linkage analysis was performed using the NPLall statistic, as implemented in MERLIN. In this method, identity by descent (IBD) probabilities are estimated for all affected pairs across all inheritance patterns. The IBDs are used in a score statistic, which is then converted to a LOD score by the method of Kong and COX [24].

Simulations were performed to assess the statistical significance of the observed results using the program MERLIN with 1000 replicates. Datasets were simulated according to the null hypothesis of no linkage across the whole genome with the same family structures, marker map, allele frequencies and patterns of missing data as what have been used in our linkage analysis. Both parametric and non-parametric analyses were performed for each replicate with the same parameters as in the linkage analysis. The significance of linkage were defined using the rates of chance occurrence as proposed by Lander and Kruglyak's [18]: suggestive (once in a genome scan) and significant (once in 20 scans, or P<0.05). The critical region of linkage was defined as the region surrounding a linkage peak yielding a LOD score that was greater than the maximum LOD–1 in each direction.

## Supporting Information

Table S1 Result of linkage analysis on chromosome 2p14.
(DOC)

Table S2 The results of simulation.
(DOC)

Supporting Information S1 Pedigree structures of the families in the study.
(DOC)
