# Supplementary material for: Chromosome 2p14 Is Linked to Susceptibility to Leprosy
Source: PLoS One. 2012 Jan 6;7(1):e29747. doi: 10.1371/journal.pone.0029747 (PMC3253103; doi:10.1371/journal.pone.0029747)
Supplement: Table S1 — Result of linkage analysis on chromosome 2p14. (DOC) [file pone.0029747.s001.doc]

**Table S1.** Result of linkage analysis on chromosome 2p14

| Chromosome | Mark | Position（cm） | ALPHA | HLOD |
| --- | --- | --- | --- | --- |
| 2 | rs1504188 | 74.87 | 0.226 | 1.106 |
| 2 | rs10183322 | 74.92 | 0.226 | 1.11 |
| 2 | rs1520446 | 75.94 | 0.284 | 1.537 |
| 2 | rs1995584 | 76.47 | 0.292 | 1.671 |
| 2 | rs1876088 | 76.52 | 0.293 | 1.681 |
| 2 | rs10183053 | 77.12 | 0.3 | 1.797 |
| 2 | rs1483869 | 77.83 | 0.304 | 1.902 |
| 2 | rs2009142 | 78.36 | 0.307 | 1.969 |
| 2 | rs2165006 | 78.67 | 0.306 | 1.968 |
| 2 | rs1961245 | 78.68 | 0.308 | 1.975 |
| 2 | rs727878 | 79.55 | 0.347 | 2.233 |
| 2 | rs1430197 | 79.56 | 0.348 | 2.235 |
| 2 | rs1432567 | 79.73 | 0.35 | 2.254 |
| 2 | rs1030092 | 79.97 | 0.389 | 2.564 |
| 2 | rs879808 | 80.04 | 0.392 | 2.601 |
| 2 | rs1017267 | 80.11 | 0.393 | 2.622 |
| 2 | rs715271 | 80.4 | 0.394 | 2.635 |
| 2 | rs1568452 | 81.28 | 0.351 | 2.374 |
| 2 | rs12053355 | 81.7 | 0.352 | 2.386 |
| 2 | rs887941 | 82.42 | 0.352 | 2.386 |
| 2 | rs359265 | 83.12 | 0.354 | 2.408 |
| 2 | rs1512226 | 83.37 | 0.354 | 2.415 |
| 2 | rs1177264 | 83.98 | 0.357 | 2.433 |
| 2 | rs13013218 | 84.68 | 0.357 | 2.414 |
| 2 | rs992214 | 84.74 | 0.356 | 2.409 |
| 2 | rs262472 | 84.79 | 0.364 | 2.446 |
| 2 | rs890478 | 84.89 | 0.376 | 2.512 |
| 2 | rs2241160 | 86.37 | 0.388 | 2.803 |
| 2 | rs2008312 | 86.69 | 0.399 | 2.91 |
| 2 | rs268866 | 86.77 | 0.401 | 2.936 |
| 2 | rs1000756 | 89.01 | 0.45 | 3.512 |
| 2 | rs1106577 | 89.24 | 0.45 | 3.513 |
| 2 | rs7598674 | 90.37 | 0.434 | 3.323 |
| 2 | rs1443651 | 91.5 | 0.422 | 3.057 |
| 2 | rs11895938 | 91.54 | 0.421 | 3.043 |
| 2 | rs9902 | 91.89 | 0.411 | 2.901 |
| 2 | rs2002879 | 92.44 | 0.363 | 2.551 |
| 2 | rs10205487 | 92.77 | 0.365 | 2.561 |
| 2 | rs897119 | 92.78 | 0.365 | 2.561 |
| 2 | rs1986601 | 92.82 | 0.365 | 2.56 |
| 2 | rs1448927 | 93 | 0.363 | 2.536 |
| 2 | rs758062 | 93.53 | 0.369 | 2.526 |
| 2 | rs227771 | 96.17 | 0.347 | 2.081 |
| 2 | rs194242 | 97.2 | 0.291 | 1.673 |
| 2 | rs12624267 | 98.19 | 0.289 | 1.656 |
| 2 | rs1653249 | 98.4 | 0.288 | 1.648 |
| 2 | rs831547 | 98.43 | 0.288 | 1.646 |
| 2 | rs828869 | 98.64 | 0.287 | 1.629 |
| 2 | rs741418 | 100.01 | 0.276 | 1.409 |
