# Supplementary material for: Chromosome 2p14 Is Linked to Susceptibility to Leprosy
Source: PLoS One. 2012 Jan 6;7(1):e29747. doi: 10.1371/journal.pone.0029747 (PMC3253103; doi:10.1371/journal.pone.0029747)
Supplement: Table S2 — The results of simulation. (DOC) [file pone.0029747.s002.doc]

**Table S2.** The results of simulation

|  | HLOD / Non-parametric LOD | |
| --- | --- | --- |
| Model | Suggestive | Significant |
| Parametric (dominant, penetrance=1) | 0.869 | 3.033 |
| Parametric (dominant, penetrance=0.5) | 0.870 | 3.041 |
| Parametric (recessive, penetrance=1) | 0.948 | 3.148 |
| Parametric (recessive, penetrance=0.5) | 0.976 | 3.103 |
| Nonparametric | 0.880 | 3.110 |
