# Supplementary material for: Chromosome 2p14 Is Linked to Susceptibility to Leprosy
Source: PLoS One. 2012 Jan 6;7(1):e29747. doi: 10.1371/journal.pone.0029747 (PMC3253103; doi:10.1371/journal.pone.0029747)
Supplement: Supporting Information S1 — Pedigree structures of the families in the study. (DOC) [file pone.0029747.s003.doc]

**Supporting information S1 Pedigree structures of the families in the study.** All investigated individuals are

marked with a triangle. clinical subtype of the disease are shown below each individual.


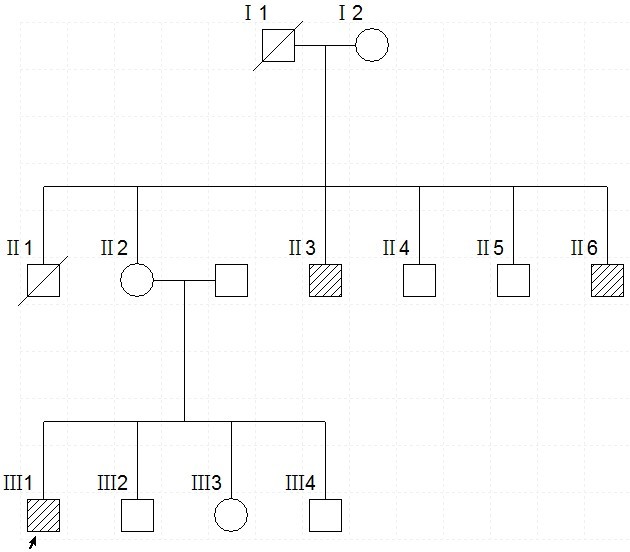


F4


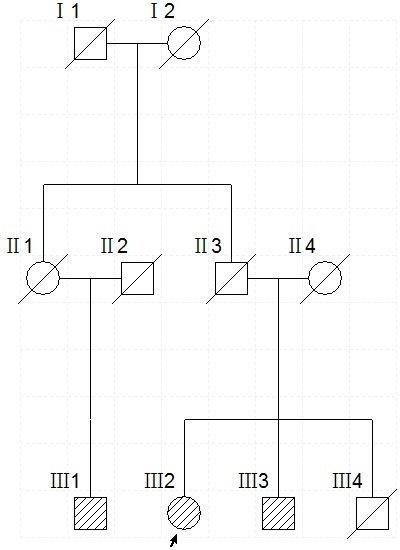


F6


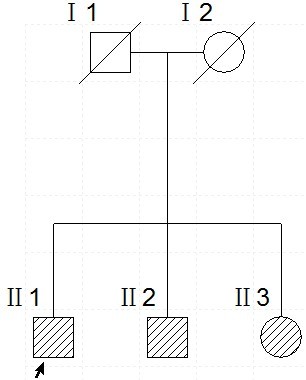


F5


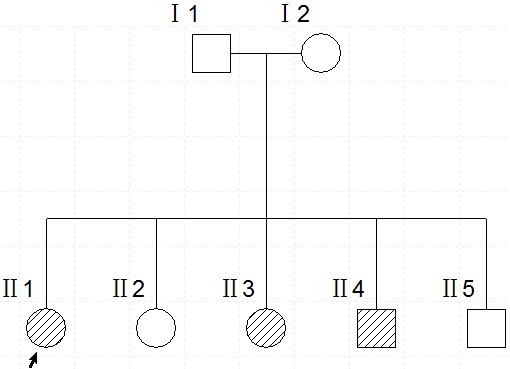


F7


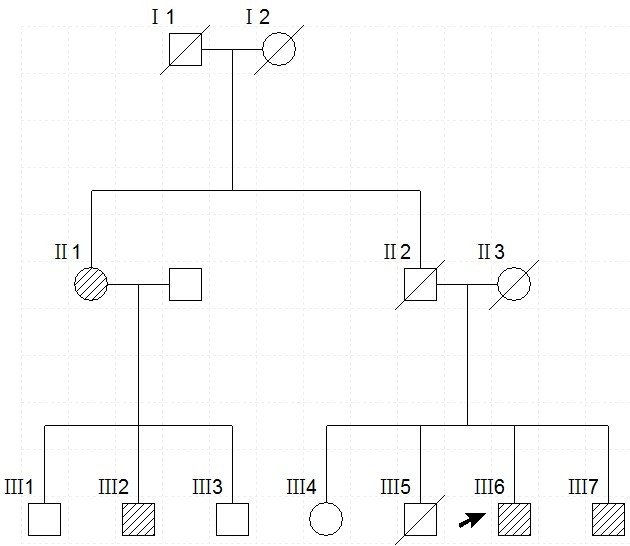


F1

F2


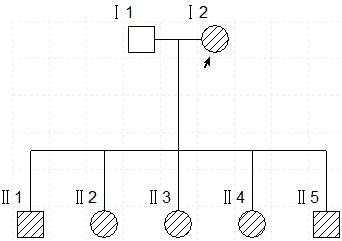


F3


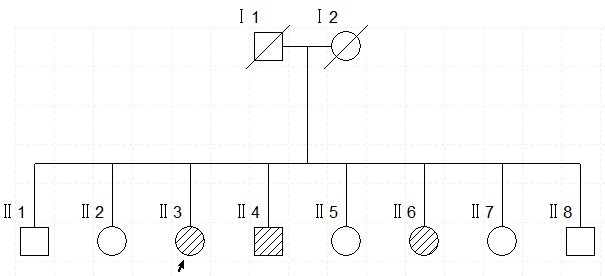

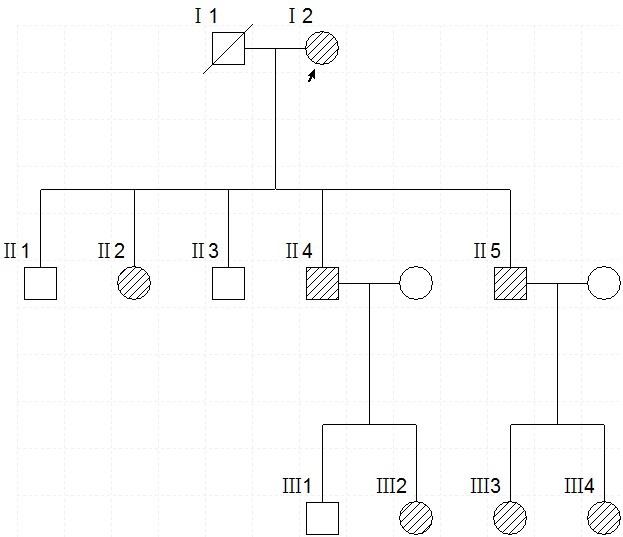


F11


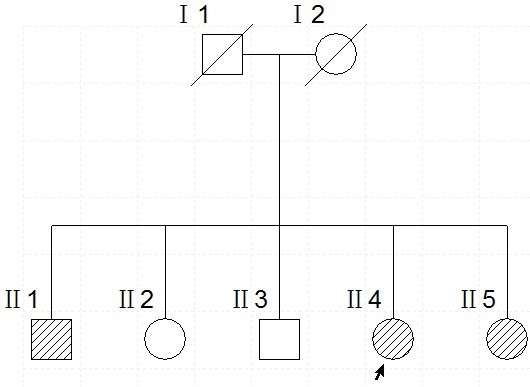


F10


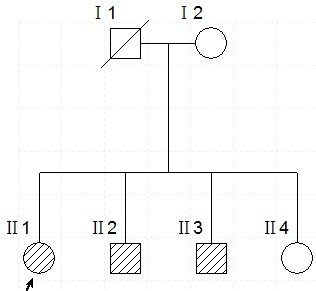


F9


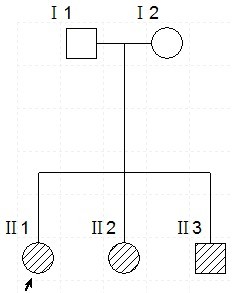


F8

LL

LL

TT

TT

BL

LL BL BT BT

LL BL BT

BL BT

TT

BL TT LL

LL BT LL

LL TT LL

BL

BL TT BT

TT BT TT

TT TT TT

BL BT BL

BT BL BT


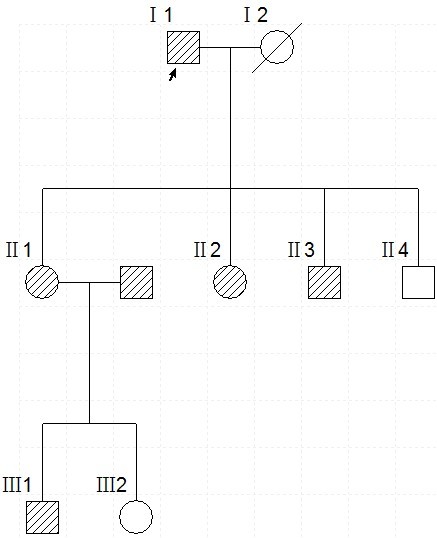


F12

F13


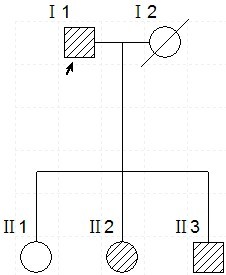

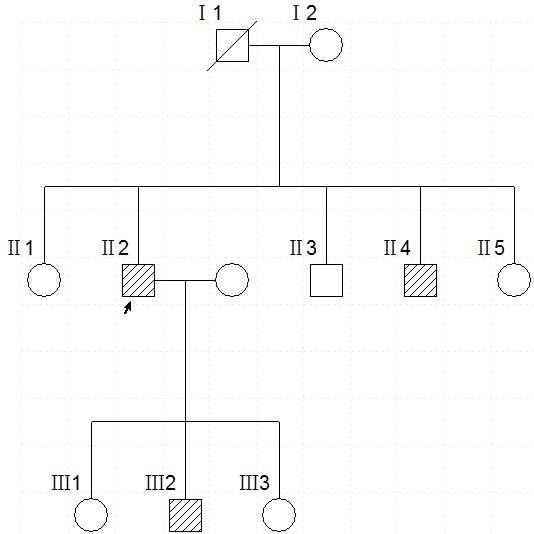


F14

F15


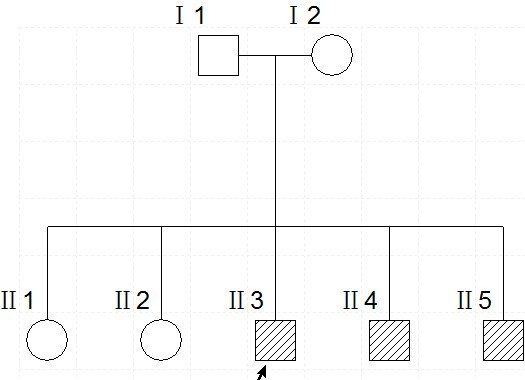


F20


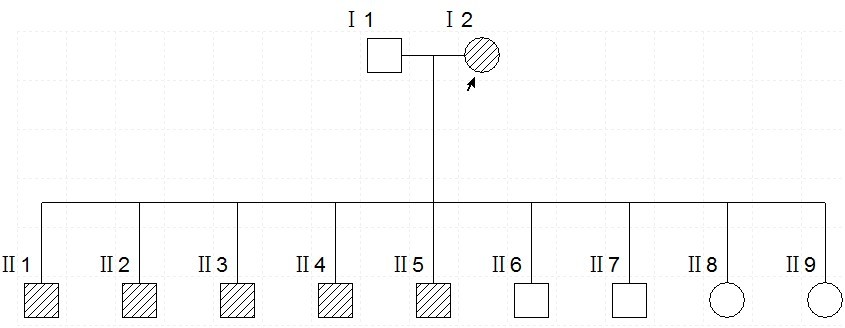


F21


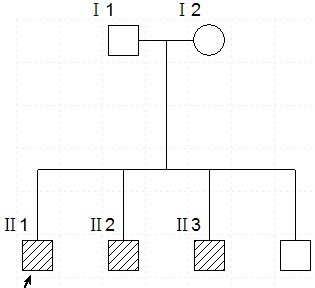

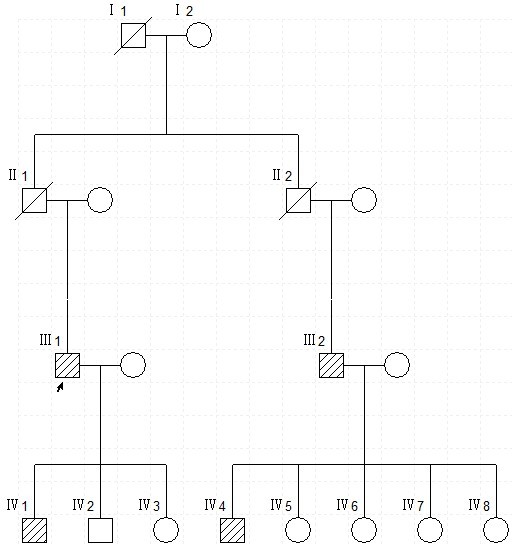


F22


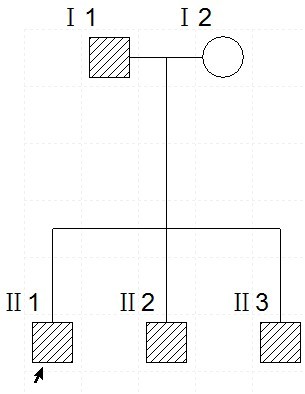


F23

F16


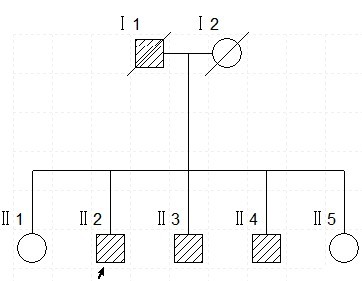


F18


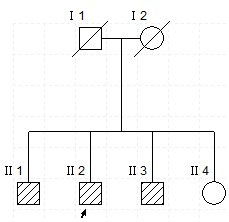

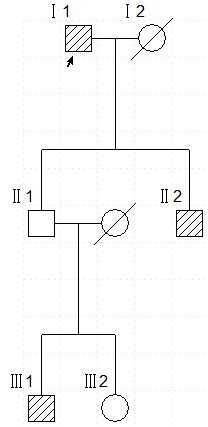


F19


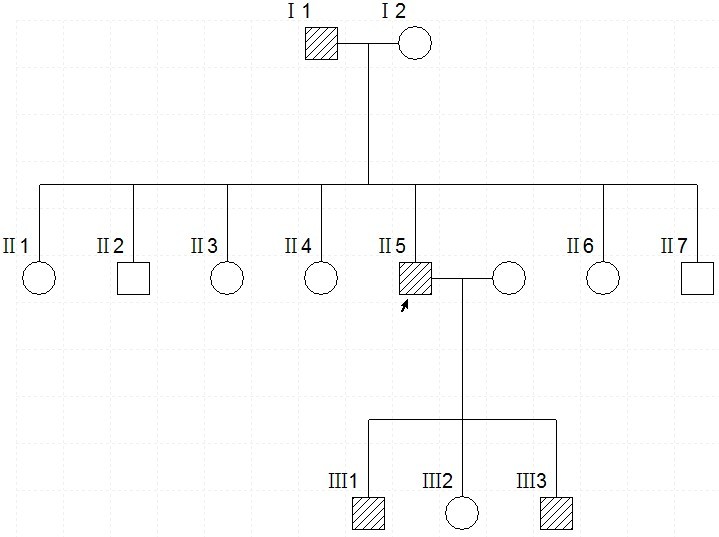


F17

BL

BL BT

BL

BL BL

BT

BB BT

LL

BL BL BL

LL

BB

BL

BB

BL BB BT

BL

LL

BT BT BL

BL

BT BL BT

LL

BB BL BT BT

BL

TT

BT BT

TT

BL BT BT
